# Gut microbiota changes related to *Helicobacter pylori* eradication with vonoprazan containing triple therapy among adolescents: a prospective multicenter study

**DOI:** 10.1038/s41598-020-80802-3

**Published:** 2021-01-12

**Authors:** Toshihiko Kakiuchi, Kentaroh Yamamoto, Ichiro Imamura, Kazutoshi Hashiguchi, Hiroharu Kawakubo, Daisuke Yamaguchi, Yasuhiko Fujioka, Masumi Okuda

**Affiliations:** 1 Department of Pediatrics, Faculty of Medicine, Saga University, Saga, Japan; 2 Department of Gastroenterology, Yamamoto Memorial Hospital, Imari, Japan; 3 Department of Gastroenterology, Imamura Hospital, Tosu, Japan; 4 Department of Gastroenterology, ImariArita Kyoritsu Hospital, Nishimatsuura, Japan; 5 Department of Gastroenterology, Ureshino Medical Center, Ureshino, Japan; 6 Department of Gastroenterology, Fujioka Hospital, Saga, Japan; 7 Department of Pediatrics, Hyogo College of Medicine, Nishinomiya, Japan

**Keywords:** Microbiology, Gastroenterology

## Abstract

Currently, it is unclear whether treating *Helicobacter pylori* (*H. pylori*) infection is safe among adolescents. This study aimed to evaluate the safety of *H. pylori* eradication therapy by examining gut microbiota changes in adolescents 3 months after the therapy. *H. pylori*-infected adolescents were enrolled in this study. Their stool samples were collected at the following three time points: before treatment, 1–2 days after completion of treatment, and time of eradication successful judgment. We assessed the relative abundance, alpha-diversity, and beta-diversity of the gut microbiota and adverse events. The number of isolated *Actinobacteria* decreased immediately after eradication therapy in the 16 students included in the study, and it returned to pretreatment condition at the eradication judgment point. There was no change in the relative abundance at genus level. The alpha-diversity was lost immediately after eradication therapy; however, it recovered at the time of eradication judgment, and it was restored to pretreatment condition. Meanwhile, none of the participants experienced serious adverse events. *H. pylori* eradication therapy is safe for adolescents with respect to gut microbiota changes associated with *H. pylori* eradication therapy. Therefore, further long-term evaluations of gut microbiota changes following eradication therapy are warranted.

## Introduction

*Helicobacter pylori* (*H. pylori*) infection is generally established at the age of ≤ 5 years in Japanese adolescents^1^, and it causes atrophic gastritis in childhood^2–5^. *H. pylori* eradication therapy is effective in preventing gastric cancer if initiated immediately after infection. Because *H. pylori* eradication reduces the damage caused to the gastric mucosa^6^, it can reduce the risk of developing gastric cancer at adulthood^7^. Considering that the current infection route of *H. pylori* is person-to-person transmission, particularly within the same family^1,8^, young individuals must take measures against *H. pylori* infection from the viewpoint of preventing this infection in the next-generation. Considering these circumstances, as a new method of preventing gastric cancer, the screening and treatment of *H. pylori* infection among young individuals have been initiated as a primary prophylactic measure in Japan^9–12^.

In Japan, triple therapy with proton-pump inhibitor (PPI), amoxicillin, and clarithromycin is an effective first-line treatment for *H. pylori* infection in children^13^. In Japan, potassium-competitive acid blocker (P-CAB) may be used instead of PPI in adults^7,14,15^, and P-CAB may be used because of high frequency of CAM-resistance *H. pylori*, even in adolescents^9,12,16^. To date, *H. pylori* eradication therapy has no serious side effects in children^9,12,16,17^; however, there is a need to conduct a long-term evaluation. Therefore, the safety of *H. pylori* eradication therapy using PPI or P-CAB in adolescents remains controversial.

Sustained infection of *H. pylori* decreases and/or increases gastric acid secretion, which might affect the gastric microbiota in adults and children. Several reports have suggested that *H. pylori* infection significantly affects the intestinal microbiota^18–21^. Antibiotic agents administered for *H. pylori* are known to quantitatively and qualitatively alter the human gut microbiota^22,23^. It has been reported that, even in children, dysbiosis is directly linked to various systemic conditions such as allergic diseases, autism spectrum disorders, and inflammatory bowel diseases^24–28^. Considering that the antibiotic agents used to eradicate *H. pylori* can affect the intestinal microbiota, it is necessary to study the safety of eradication therapy for *H. pylori*. A previous study reported the effect of probiotics during vonoprazan-containing triple therapy on the intestinal microbiota in individuals with *H. pylori* infection^29^; however, the study only examined the intestinal microbiota before and immediately after eradication therapy. Thus, this study aimed to evaluate the safety of *H. pylori* eradication therapy by examining the gut microbiota changes 3 months after therapy in adolescents.

## Results

**Figure 1:**
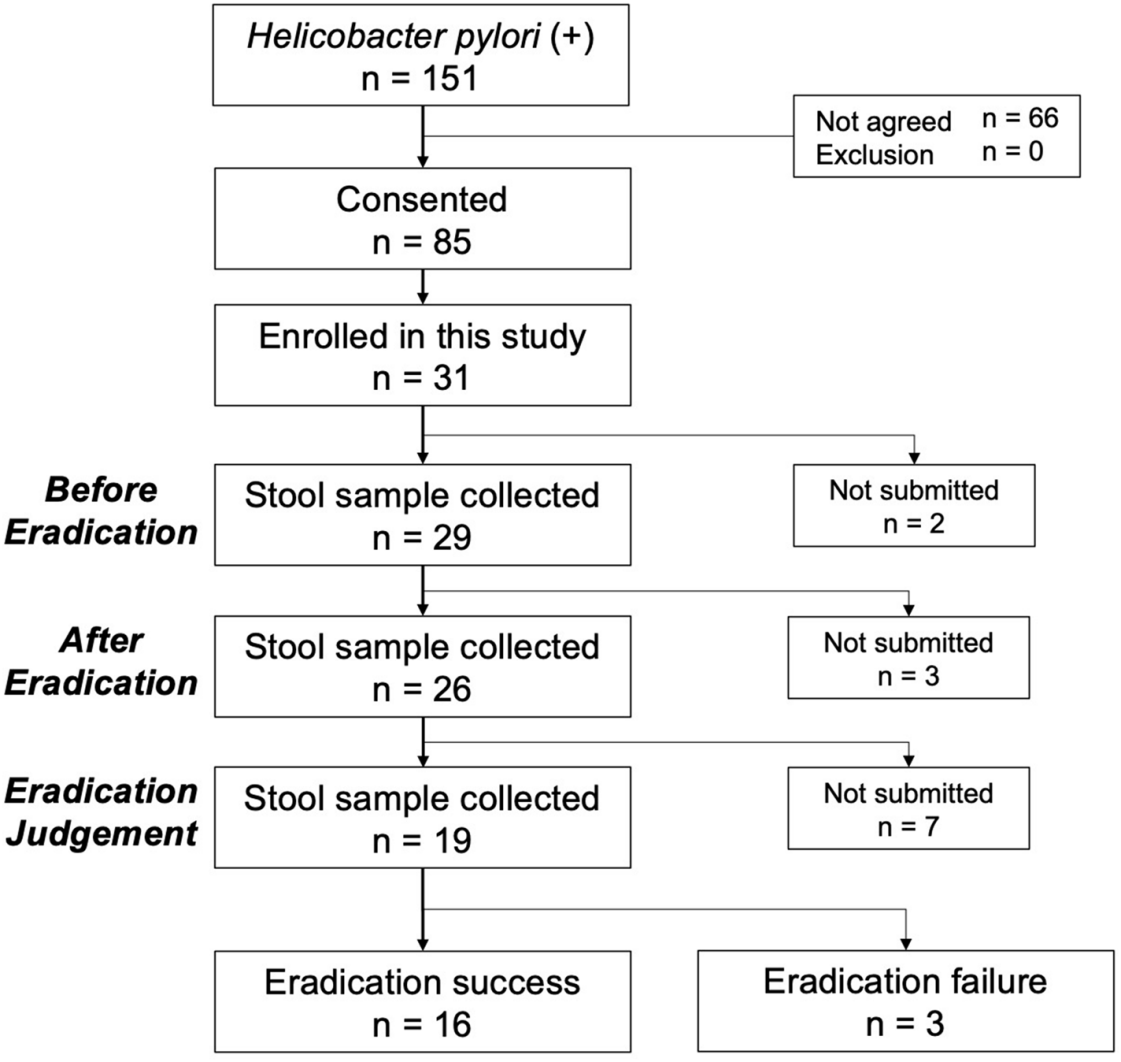
Flow chart of patient enrollment.

We enrolled students with two positive test results for *H. pylori* between June 2017 and March 2019. The flow of patient enrolment is presented in Fig. 1. Among the 151 patients with an *H. pylori* infection, 31 provided consent and participated in this study. There were 29 students who provided stool samples before eradication, 26 immediately after eradication, and 19 at the time of eradication judgment. Of these 19 patients, 3 experienced primary treatment failure; therefore, 16 (n = 9, male, n = 7, female; median age: 15.6 [15.1–15.9] years) were considered eligible for this study. This study was conducted as a sub analysis of *H. pylori* infection screening of junior high school third-year students in Saga Prefecture. Considering this, we could not thoroughly examine the background of the students. These students submitted their stool samples at each of the three time points. The *H. pylori* eradication assessments were conducted on median day 92.5 (range 89–107 days) after the eradication therapy, and the stool samples were collected on the median day 99 (range 91.5–108.5 days).

### Relative abundance

**Figure 2:**
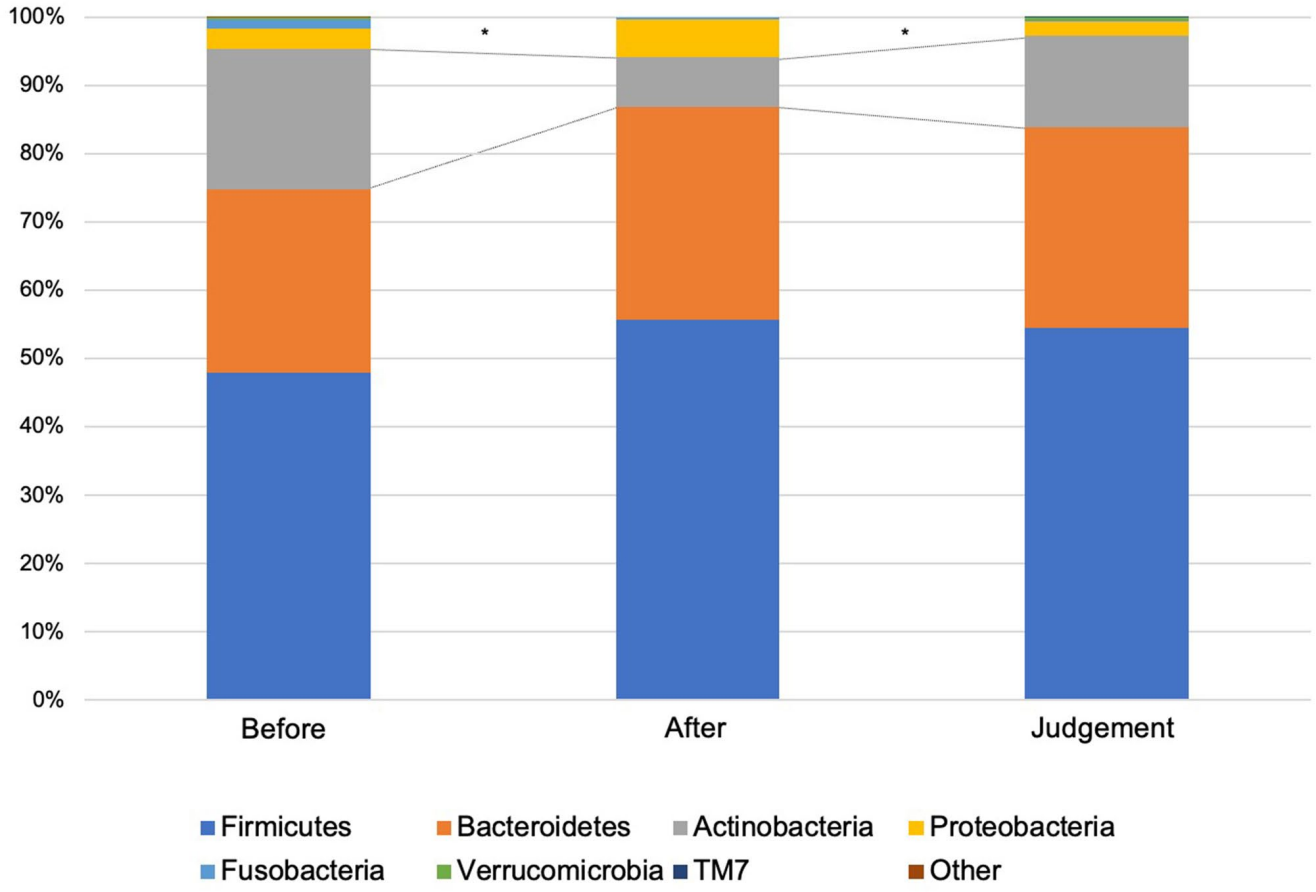
Relative abundance of the intestinal microbiota at the phyla at the following three time points: before eradication therapy, after eradication therapy, and at the time of eradication therapy judgment.

**Figure 3:**
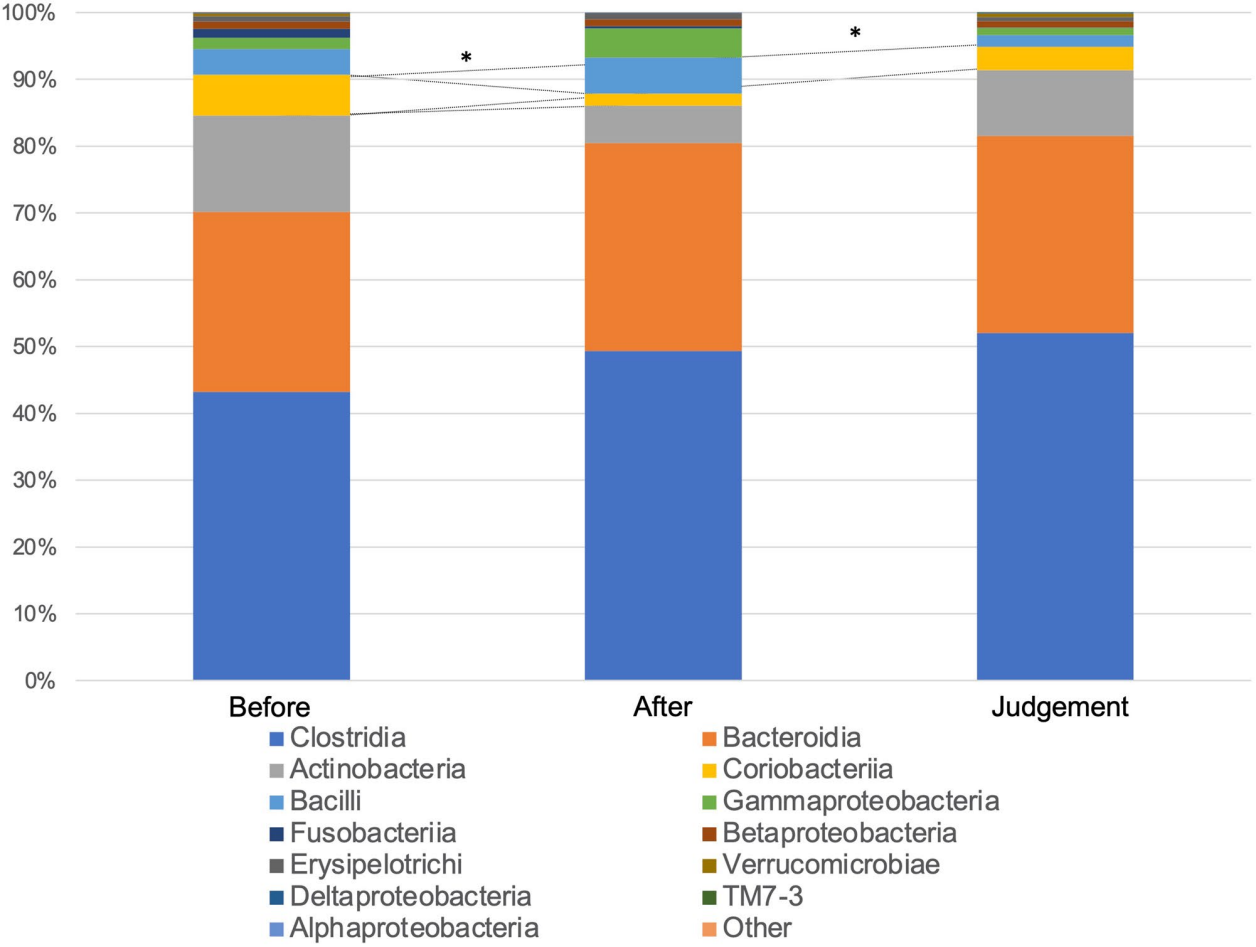
Relative abundance of the intestinal microbiota at the class level at the following three time points: before eradication therapy, after eradication therapy, and at the time of eradication therapy judgment.

**Figure 4:**
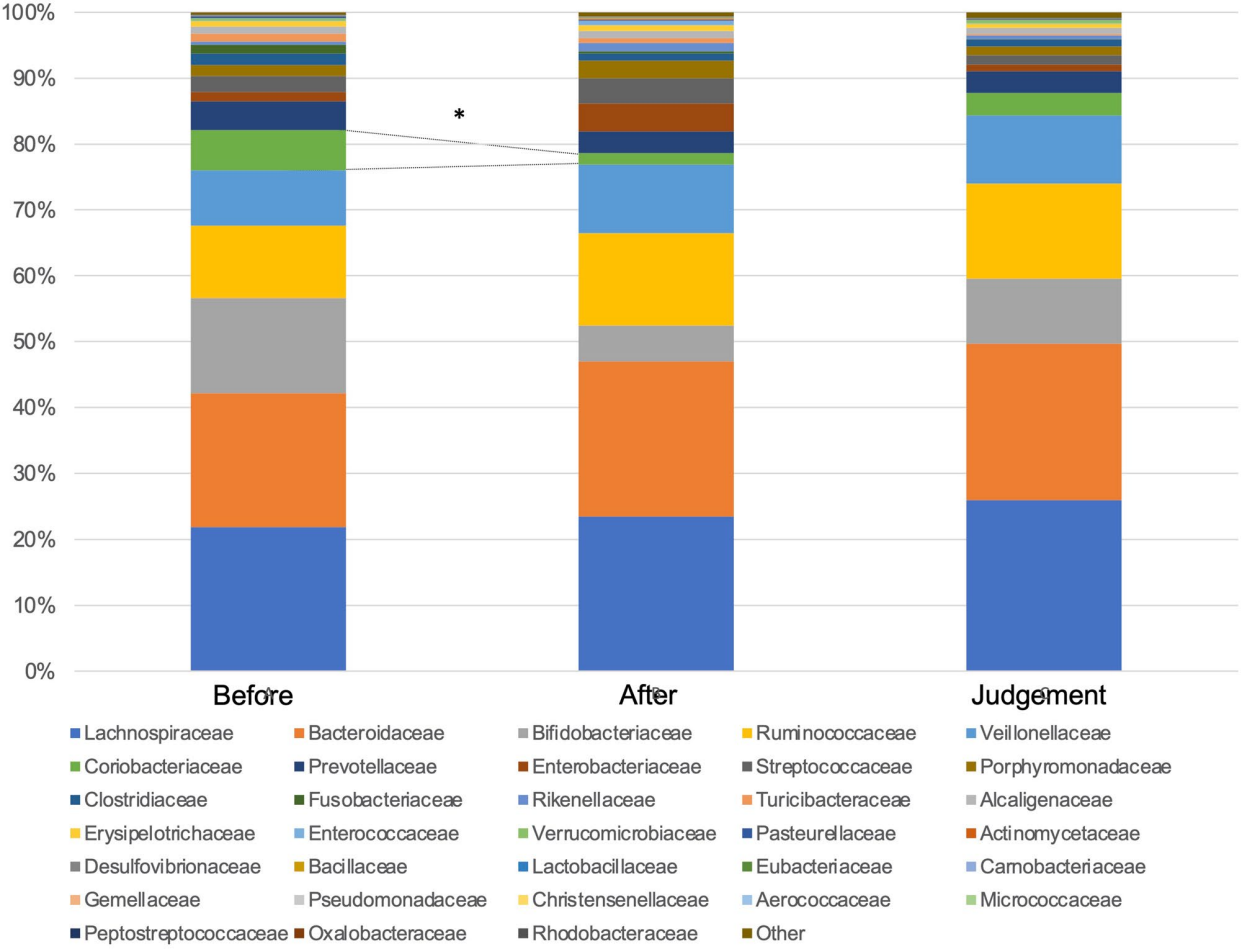
Relative abundance of the intestinal microbiota at the family level at the following three time points: before eradication therapy, after eradication therapy, and at the time of eradication therapy judgment.

**Figure 5:**
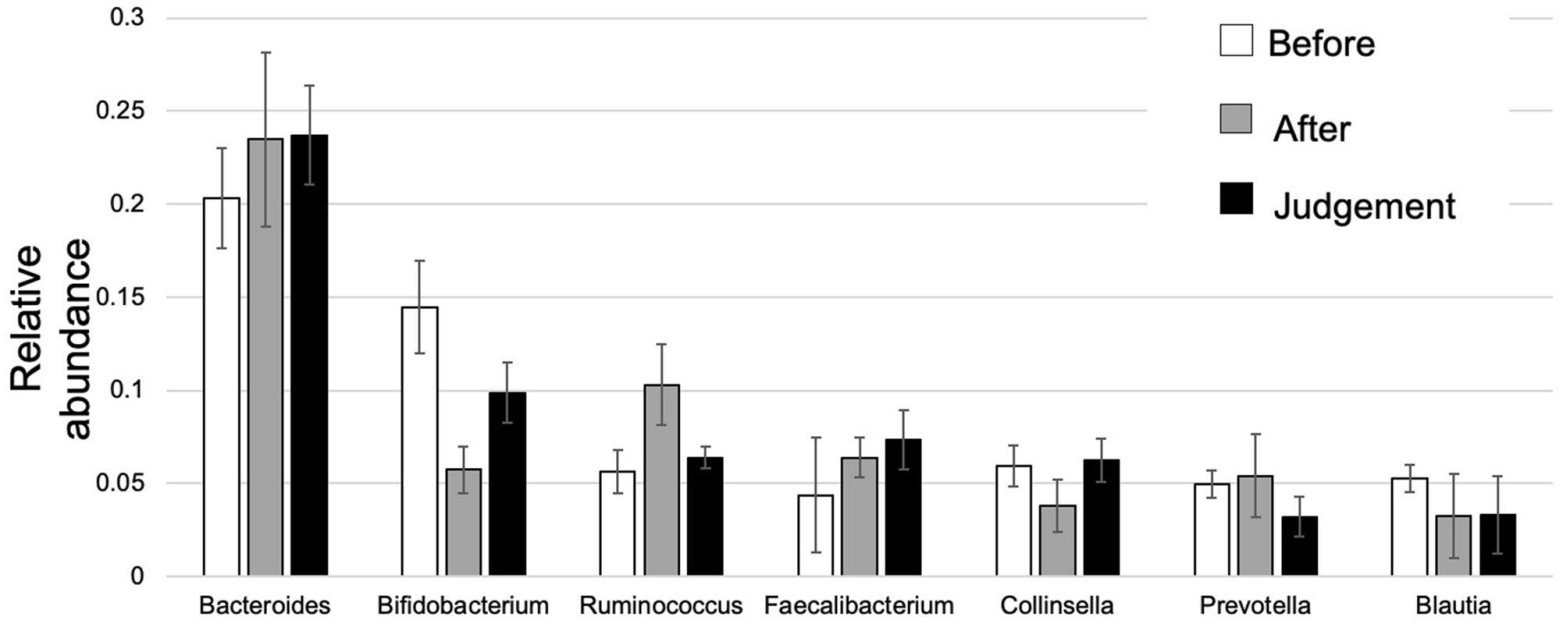
Relative abundance of the intestinal microbiota larger than 3% at the following three time points: before eradication therapy, after eradication therapy, and at the time of eradication therapy judgment.

Figure 2 shows the compositions and relative abundance of the gut microbiota at the phyla level. The proportion of *Actinobacteria* significantly decreased after eradication therapy compared with before eradication therapy (*P* = 0.006924). On the contrary, the proportion of *Actinobacteria* significantly increased at the time of eradication assessment compared with after eradication therapy (*P* = 0.047065), and no significant difference was observed between before eradication therapy and at the time of eradication assessment (*P* = 0.214554). The compositions and relative abundance of the gut microbiota at the class level are shown in Fig. 3. Compared with before eradication therapy, the proportion of *Coriobacteriia* significantly decreased after eradication therapy (*P* = 0.003564) and eradication assessment (*P* = 0.025209). The compositions and relative abundance of the gut microbiota at the family level are shown in Fig. 4. Compared with before eradication therapy, the proportion of *Coriobacteriaceae* significantly decreased after eradication therapy (*P* = 0.008656). Figure 5 shows the top seven genus compositions of the relative abundance of gut microbiota. In each of the seven genuses, no significant changes were observed in the comparison results before eradication therapy, after eradication therapy, and during eradication judgment.

### Alpha-diversity

**Figure 6:**
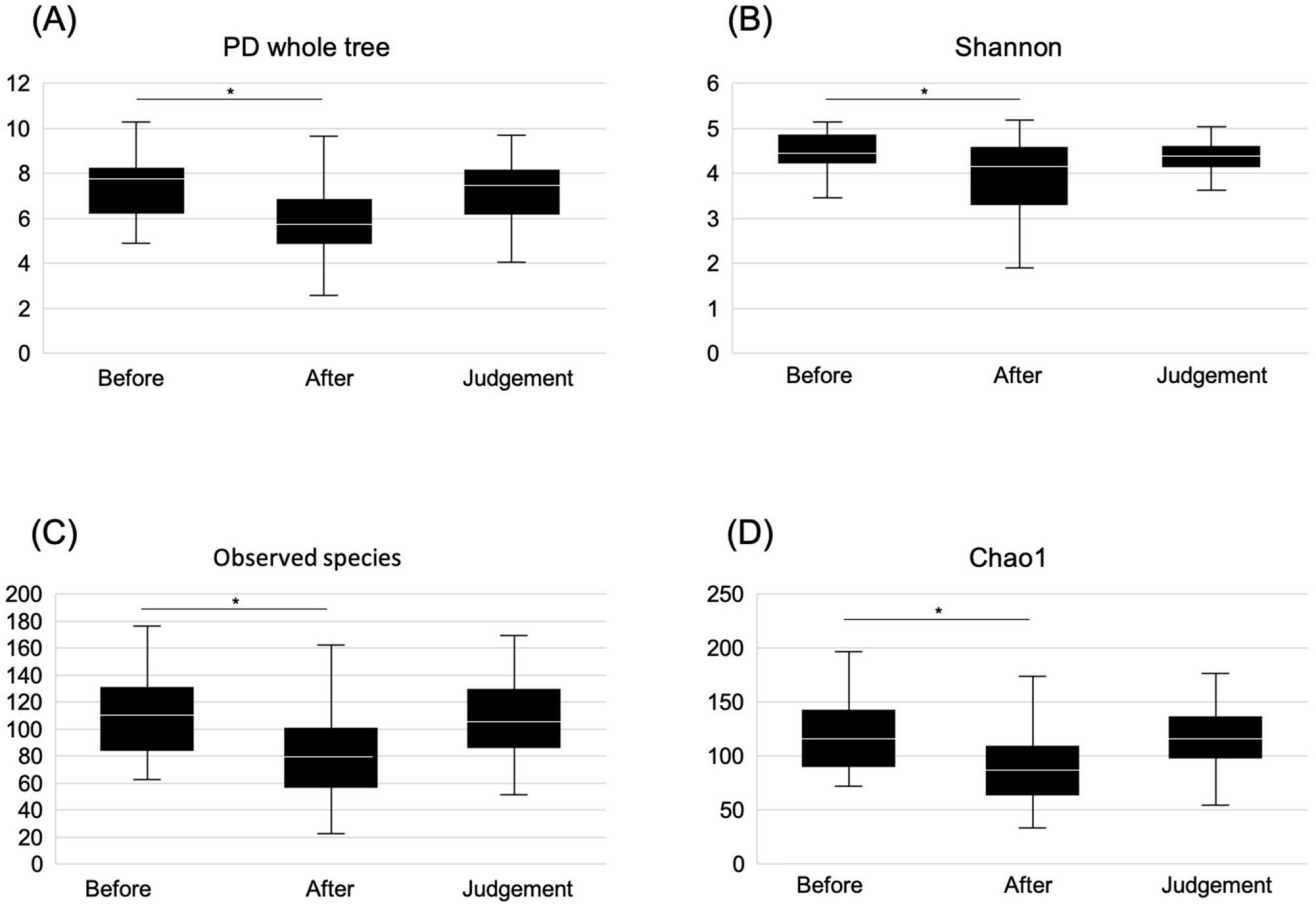
Boxplot depicting α-diversity before eradication therapy, after eradication therapy, and at the time of eradication judgment. (**A**) PD whole tree, (**B**) Shannon index, (**C**) Observed OTUs, and (**D**) PD whole tree. Comparison before and after eradication therapy (PD whole tree, *P* = 0.003; Shannon index, *P* = 0.023; observed OTUs, *P* = 0.003; and Chao1, *P* = 0.001). Comparison between before eradication therapy and at the time of eradication therapy judgment (PD whole tree, *P* = 1.000; Shannon index, *P* = 1.000; observed OTUs, *P* = 1.000; and Chao1, *P* = 1.000). Comparison between after eradication treatment and at the time of eradication therapy judgment (PD whole tree, *P* = 0.063; Shannon index, *P* = 0.492; observed OTUs, *P* = 0.106; and Chao1, *P* = 0.061).

We calculated the α-diversity according to the PD whole tree, Shannon index, observed OTUs, and Chao1 at the three time points. Phylogenetic richness (PD whole tree; *P* = 0.003), diversity of microbiota (Shannon index; *P* = 0.023), and microbial species richness (observed OTUs; *P* = 0.003, Chao1; *P* = 0.001) after eradication therapy were significantly lower than those before eradication therapy. However, there were no significant differences between the PD whole tree (*P* = 1.000), Shannon index (*P* = 1.000), observed OTUs (*P* = 1.000), and Chao1 (*P* = 1.000) at the time of eradication judgment and those before eradication therapy. Moreover, the PD whole tree (*P* = 0.063), Shannon index (*P* = 0.492), observed OTUs (*P* = 0.106), and Chao1 (*P* = 0.061) at eradication judgment point did not significantly differ from those after eradication therapy (shown in Fig. 6).

### Beta-diversity

**Figure 7:**
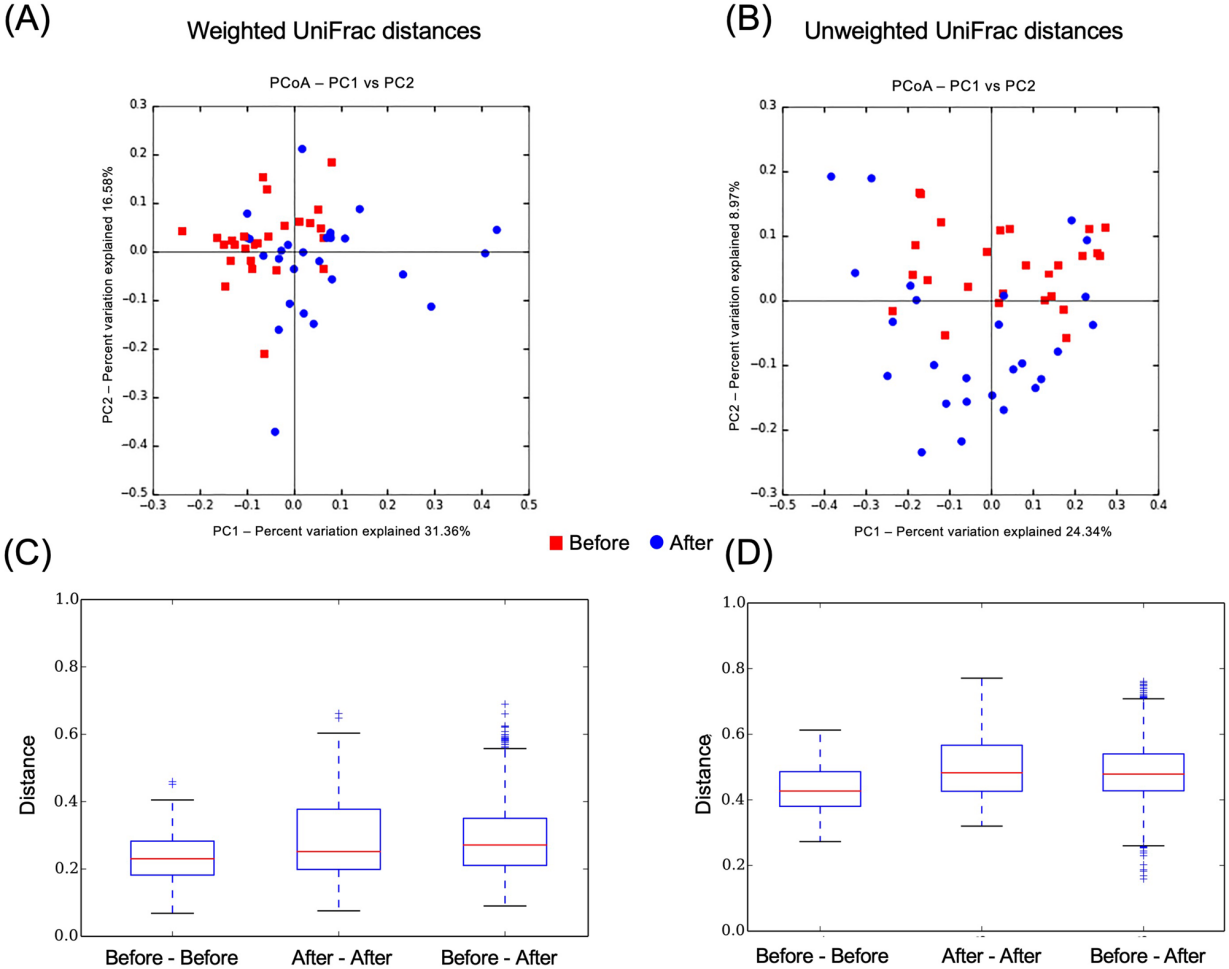
Principal coordinates analysis revealed clustered communities of gut microbiota after eradication in the before and after eradication therapy groups. (**A**) Weighted UniFrac distances, (**B**) Unweighted UniFrac distances. Bar diagram showing the Mean UniFrac distances for Before–Before, After–After, and Before–After participants. (**C**) Weighted UniFrac distances (Before–Before vs. Before–After, *P* = 0.03; After–After vs. Before–After, *P* = 1.00), (**D**) Unweighted UniFrac distances (Before–Before vs. Before–After, *P* = 0.03; After–After vs. Before–After, *P* = 0.98).

**Figure 8:**
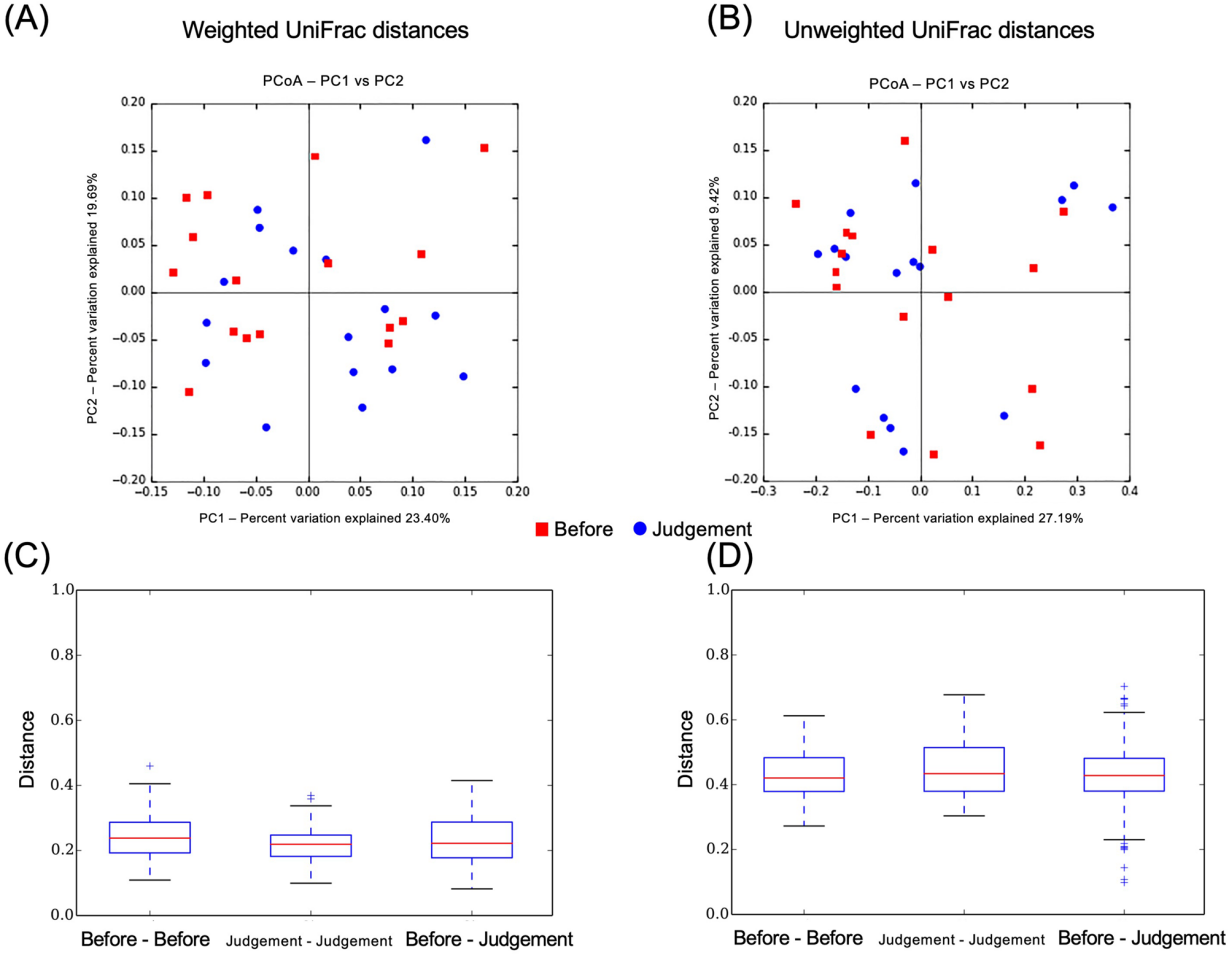
Principal coordinates analysis revealed clustered communities of gut microbiota after eradication in the before and after eradication therapy groups. (**A**) Weighted UniFrac distances, (**B**) Unweighted UniFrac distances. Bar diagram showing the Mean UniFrac distances for Before–Before, Judgment–Judgment, and Before–Judgment participants. (**C**) Weighted UniFrac distances (Before–Before vs. Before–Judgment, *P* = 1.00; Judgment–Judgment vs. Before–Judgment, *P* = 1.00), (**D**) Unweighted UniFrac distances (Before–Before vs. Before–Judgment, *P* = 1.00; Judgment–Judgment vs. Before–Judgment,* P* = 0.87).

The β-diversity, calculated based on the weighted and unweighted UniFrac distances, revealed that the gut microbiota structures were similar before and after eradication therapy (weighted UniFrac distances: Before–Before vs. Before–After, *P* = 0.03; After–After vs. Before–After, *P* = 1.00. unweighted UniFrac distances: Before–Before vs. Before–After, *P* = 0.03; After–After vs. Before–After, *P* = 0.98) (shown in Fig. 7). Similarly, the β-diversity revealed that the gut microbiota structures were similar between before eradication therapy and judgment of eradication therapy (weighted UniFrac distances: Before–Before vs. Before–Judgment, *P* = 1.00; Judgment–Judgment vs. Before–Judgment, *P* = 1.00. unweighted UniFrac distances; Before–Before vs. Before–Judgment, *P* = 1.00; Judgment–Judgment vs. Before–Judgment, *P* = 0.87) (shown in Fig. 8).

### Adverse events

Among the 16 patients 1 (6.35%) presented with diarrhea and 2 (12.5%) presented with abdominal pain. None of the adverse events were serious. Therefore, therapy was not required, and treatment for the eradication of *H. pylori* was not interrupted.

## Discussion

The present study revealed changes in the gut microbiota before and after *H. pylori* eradication therapy using 16S rRNA gene/DNA/amplicon sequencing with next-generation sequencing and amplicon analysis in Japanese adolescents. Two important clinical suggestions were obtained. First, change in the gut microbiota during eradication therapy of *H. pylori* with vonoprazan fumarate containing triple therapy in adolescents led to dysbiosis immediately after eradication therapy; however, it returned to pretreatment conditions 3 months after the eradication therapy (shown in Figs. 2 and 6). Second, the eradication therapy of *H. pylori* with vonoprazan fumarate containing triple therapy was found safe for children from the perspective of adverse events.

Yap TW et al. reported that the eradication of *H. pylori* caused perturbation of the gut microbiota, and it may indirectly affect the health of the patients^30^. By contrast, Gotoda T et al. reported that although *H. pylori* eradication therapy caused short-term dysbiosis, microbial diversity was restored in healthy Japanese adolescents^31^. To the best of our knowledge, this was the first study to evaluate the changes in the gut microbiota before and after *H. pylori* eradication therapy in adolescents. Moreover, α-diversity analysis revealed that species microbial richness and evenness were recovered to pretreatment levels at 2 months after eradication therapy. The proportion of *Actinobacteria* also significantly decreased immediately after eradication therapy, and it recovered similarly 2 months after eradication. Our results were considerably similar to those of a previous study (shown in Figs. 2 and 6)^31^. Cornejo-Pareja et al. observed a significant decrease in the number of *Actinobacteria* after the administration of omeprazole, clarithromycin, and amoxicillin in patients with *H. pylori* infection^32^. Our results also showed a decrease in *Actinobacteria*, which was due to a decrease in Coriobacteriia and Coriobacteriaceae at the class and family levels, respectively. We assumed that this occurred due to the effect of drug susceptibility to the antibiotics used in the eradication therapy, but we are unsure of the exact etiology. In the present study, the safety of eradication therapy among adolescents may be recommended with respect to gut microbiota because the dysbiosis of gut microbiota recovered after several months to pretreatment levels. Liou JM et al. reported that although the eradication therapy for *H. pylori* infection causes minimal disruption in the microbiota, their results support the long-term safety of *H. pylori* eradication therapy^33^.

Several studies showed that *H. pylori* infection in children caused the changes in the gut microbiota^18,34–36^. The diversity of intestinal microbiota is particularly reduced in adults with *H. pylori* infection^37^. Benavides-Ward et al. reported that children with *H. pylori* infection presented with an increase in the number of bacteria, such as *Proteobacteria*, *Clostridium*, *Firmicutes*, and *Prevotella*, in the gut microbiota^34^. However, information related to the relationship between *H. pylori* infection and intestinal bacteria included in the gut microbiota is still limited. Owing to the presence of differences in gut microbiota between *H. pylori*-infected and non-infected children, it cannot be denied that *H. pylori* infection affects children. From this perspective as well, *H. pylori* eradication in children appears to be necessary. To confirm the long-term safety of *H. pylori* eradication, future studies must be conducted to directly compare gut microbiota between the children who have remained healthy for a long time after *H. pylori* eradication therapy and healthy children without *H. pylori* infection.

The adverse events observed in our study included diarrhea and abdominal pain. However, these were not serious adverse events. The safety of eradication therapy of *H. pylori* with triple therapy containing vonoprazan fumarate for adolescents remains unclear; however, reports have shown no major problems in short-term evaluations^9,12,16^. In the present study, the number of cases was limited, and only adverse events during oral administration of antibacterial drugs and P-CAB were evaluated. Therefore, future studies with a larger sample size are warranted.

In Japan, the screening and treatment of *H. pylori* infection among adolescents are performed as a primary preventive measure against gastric cancer. By contrast, the clinical practice guidelines do not recommend screening and treatment for asymptomatic *H. pylori*-infected children to prevent gastric cancer^38,39^. However, these guidelines are for children and adolescents living in Europe and North America, and they may not be applicable to those living in other continents, particularly in developing countries with a high *H. pylori* infection rate and limited health care resources^40^. The safety of *H. pylori* screening and treatment for adolescents is debated in Japan^13^. According to the guidelines for the management of *H. pylori* infection in childhood, *H. pylori* eradication therapy for children younger than 15 years is not recommended owing to safety concerns. Our study showed that dysbiosis developed immediately after *H. pylori* eradication therapy (shown in Figs. 2 and 6). However, it returned to pretreatment condition after 3 months. This may be one factor for the safety of *H. pylori* eradication treatment. To prevent gastric cancer, *H. pylori* infection must be eradicated at a young age^6^. However, from the viewpoint of gastric cancer prevention, it is controversial whether the ideal period of *H. pylori* eradication is childhood or adulthood. It is certain that gastric mucosal atrophy associated with *H. pylori* infection is a high risk of well-differentiated gastric cancer^41^. Furthermore, gastric mucosal atrophy is known to occur since childhood^42–45^, and a small proportion of gastric cancers that occur in childhood^46^. Therefore, it seems unsafe to conclude that eradication in adulthood is absolutely sufficient to prevent gastric cancer. Although the risk of *H. pylori* reinfection after eradication therapy must also be considered, the reinfection rate is considered to be extremely low^47–49^. Moreover, the safety of *H. pylori* eradication therapy for adolescents must be further validated^7,50^.

This study had several limitations. First, the sample size was relatively small. Second, this study did not include placebo treatments for comparison. Third, the altered intestinal microbiota could be evaluated; the functionality of the bacteria itself and how it affected humans were also not evaluated. Intestinal microbiota analysis could not be performed by further analysis methods, such as UPGMA, LEfSe, and KEGG pathway. Fourth, because diet was important for microbiota evaluation, it was not possible to grasp the meal contents of each participant. However, because they were Japanese adolescents of almost the same age living in a single prefecture, it was presumed that there would be no major dietary differences.

*H. pylori* eradication therapy may be safe for adolescents with respect to gut microbiota. Therefore, further long-term evaluations of the changes in gut microbiota after eradication therapy must be performed. If the safety of *H. pylori* eradication therapy in adolescents is validated, it will promote screening and treatment among adolescents to prevent gastric cancer.

## Materials and methods

### Study design and participants

The present study performed a sub analysis of *H. pylori* infection screening as part of the junior high school health screening system among third-year students in Saga Prefecture^9^. Using local governmental grants, a screening and treatment program for eradicating *H. pylori* infection among third-grade junior high students was started in Saga Prefecture in 2016. The students underwent urinary anti-*H. pylori* antibody tests (RAPIRAN; Otsuka Pharmaceutical Co., Ltd. Tokyo, Japan), followed by *H. pylori* stool antigen tests (TESTMATE RAPID RYLORI ANTIGEN; Wakamoto Pharmaceutical Co., Ltd. Tokyo, Japan). To eradicate *H. pylori* infection, those who tested positive on both tests received triple therapy comprising 20 mg vonoprazan fumarate (Takeda Pharmaceutical Co., Ltd. Tokyo, Japan), 750 mg amoxicillin, and 200 mg clarithromycin twice a day for 7 days. No probiotics were added to the eradication treatment nor were they used during the subsequent follow-up period. Then, 8–12 weeks after the eradication therapy, ^13^C-urea breath test (^13^C-UBT) was conducted at one of the cooperating medical institutions to assess for treatment efficacy. Breath samples were obtained 4 h after a meal and 20 min after the ingestion of 100 mg ^13^C-urea (UBIT tablet, 100 mg). An infrared spectrometer (POC ONE; Otsuka Electronics Co., Ltd., Hirakata, Japan) was used for the test (negative ≤ 2.5%).

Students were excluded from receiving eradication therapy if they were allergic to any study drugs, had impaired liver or kidney function, were receiving colchicine, had infectious mononucleosis (or suspected infection with Epstein–Barr virus), or weighed < 30 kg. Students who had taken antibiotics or antacids (PPI, P-CAB, and histamine H2-receptor antagonist) within a year were excluded because of their effects on gut microbiota. The stool samples were collected prior to treatment (Before), 1–2 days after completion of treatment (After), and after 8–12 weeks of eradication therapy when it was determined that *H. pylori* had been eradicated (Judgment). At the time of judgment of eradication therapy, students with unsuccessful treatment outcomes were excluded to rule out the effect of the presence of *H. pylori* on the gut microbiota^34^. The outcome of *H. pylori* eradication therapy was confirmed using the ^13^C-UBT. The stools were immediately stored at − 20 °C until DNA extraction. Adverse events associated with the eradication therapy were collected through a self-report questionnaire during the eradication treatment and by interview with the attending physician during the follow-up period from the eradication treatment at ^13^C-UBT for eradication judgment.

The institutional review board of Saga University Hospital (approval numbers: 2017-03-01, approval date: June 5, 2017) approved the present study. As a sub analysis of the previous studies^13^, we examined the gut microbiota changes 3 months after therapy related to *H. pylori* eradication with vonoprazan fumarate containing triple therapy among adolescents. All methods were carried out in accordance with the relevant guidelines and regulations or the guidelines of the Declaration of Helsinki. Written informed consent was obtained from all participants and their parents or guardians.

### DNA extraction

The specimens were stored at − 20 °C until DNA extraction. Bacterial DNA was extracted using the NucleoSpin Microbial DNA kit (MACHERY-NAGEL, Düren, Germany) according to the manufacturer’s instructions. The total DNA was eluted in 50 μL of elution buffer and was stored at − 20 °C. The V3–V4 hypervariable regions of 16S ribosomal DNA (rDNA) were amplified using the 16S (V3–V4) Metagenomic Library Construction Kit for NGS (Takara Bio Inc., Kusatsu, Japan) with primer pairs (341F 5′-TCGTCGGCAGCGTCAGATGTGTATAAGAGACAGCCTACGGGNGGCWGCAG-3′, 806R 5′-GTCTCGTGGGCTCGGAGATGTGTATAAGAGACAGGGACTACHVGGGTWTCTAAT-3′). The amplicon was purified using AMPure XP magnetic beads (Beckman Coulter, Brea, CA, the USA). The index PCR was performed using the Nextera XT Index Kit (Illumina, San Diego, CA, the USA). After purification with AMPure XP beads, sequencing was conducted on a MiSeq platform with the MiSeq Reagent Kit v3 and Phix Control Kit v3 (Illumina) from Takara Bio Inc.

### 16 s rDNA sequence analysis

The 16S rRNA gene/DNA/amplicon sequencing results with next-generation sequencing were analyzed as follows: low-quality sequences were removed, chimeras were checked, operational taxonomic units (OTUs) were constructed, and taxonomy was assigned using CD-HIT-OTU, Quantitative Insights Into Microbial Ecology pipeline (http://qiime.org/). OTUs were established by clustering with a 97% identity threshold and were completed using an RDP classifier with the Green genes database. The observed OTUs, Chao1 (microbial species richness), and Shannon indices (microbial evenness) and PD whole tree (phylogenetic richness) were calculated to determine the alpha (α)-diversity of the microbiota in the samples. The beta (β)-diversity of our samples was calculated using the default beta-diversity metrics of the weighted and unweighted UniFrac distance. To compare the differences in the overall bacterial gut microbiota structure, a principal coordinates analysis was performed to reduce the dimensionality of the resulting distance.

### Statistical analysis

The raw data were expressed as percentage, mean, and standard deviation whenever applicable. For skewed data, the Mann–Whitney U test and Wilcoxon signed-rank test were used to compare variables between and within groups, respectively. The comparison of α-diversity and group distance for β-diversity was performed using the Monte Carlo two-sample test, followed by the false discovery rate and Bonferroni correction. A *P* value < 0.05 was considered statistically significant. Statistical analyses of the microbiota were performed using Python. Other statistical analyses were performed using R (R Core Team 2018]. R: A language and environment for statistical computing; R Foundation for Statistical Computing, Vienna, Austria; URL: https://www.R-project.org/).

### Study endpoint

The study endpoint was the change in the intestinal microbiota during *H. pylori* eradication with vonoprazan-containing triple therapy. In particular, the relative abundance, α-diversity, and β-diversity of the gut microbiota were compared before eradication, immediately after eradication, and during assessment of treatment efficacy. In addition, the adverse effects of *H. pylori* eradication with vonoprazan-containing triple therapy in children were evaluated.

## Abbreviations

*H. pylori*: *Helicobacter pylori*
PPI: Proton-pump inhibitor
P-CAB: Potassium-competitive acid blocker
rDNA: Ribosomal DNA
OTUs: Operational taxonomic units
